# Evolution and advances in endovascular mechanical thrombectomy of cerebral venous sinus thrombosis

**DOI:** 10.7150/ijms.99362

**Published:** 2024-09-23

**Authors:** Yuan Kan, Baoying Song, Miaowen Jiang, Yang Zhang, Chuanhui Li, Chuanjie Wu, Wenhao Zhou, Ang Li, Wenbo Zhao, Bowei Zhang, Yan Wu, Ming Li, Xunming Ji

**Affiliations:** 1 Department of Neurology and Beijing Institute of Geriatrics, Xuanwu Hospital, Capital Medical University, Beijing, 100053, China.; 2 China-America Institute of Neurology, Xuanwu Hospital, Capital Medical University, Beijing, 10069, China.; 3 Beijing Institute for Brain Disorders, Capital Medical University, Beijing, 100069, China.; 4 Shanxi Key Laboratory of Biomedical Metallic Materials, Northwest Institute for Nonferrous Metal Research, Xi'an, 710016, China.; 5 Department of Biomedical Engineering, Columbia University, New York City, NY, 10027, USA.; 6 Beijing Key Laboratory of Hypoxic Conditioning Translational Medicine, Xuanwu Hospital, Capital Medical University, Beijing, 100053, China.; 7 Department of Neurology, Massachusetts General Hospital, Harvard Medical School, Boston, MA, 02115, USA.; 8 Department of Neurosurgery, Xuanwu Hospital, Capital Medical University, Beijing, 100053, China.; Yuan Kan and Baoying Song contributed equally to this paper.

**Keywords:** Cerebral venous sinus thrombosis (CVST), Endovascular treatment (EVT), Mechanical thrombectomy (MT), Local intrasinus thrombolysis (LIST), Multimodal treatment strategies, Stent

## Abstract

Cerebral venous sinus thrombosis (CVST) is a rare type of stroke and standard treatment involves anticoagulation. However, for some special CVST patients who are ineligible for anticoagulation or refractory to conservative treatment, endovascular treatment (EVT) may be an effective option. Mechanical thrombectomy (MT) is a commonly used treatment. Compared with anticoagulation treatment alone, MT may result in additional procedure-related complications, however, many studies have shown that it has a high rate of vessel recanalization and lower incidence of related complications in arterial large vessel occlusion stroke. In addition, the applicability of MT in children, patients with deep cerebral thrombosis, and patients with bleeding before treatment has been reported. MT combined with intravascular thrombolysis (IVT) and other multimodal therapeutic strategies, also has a good curative effect, and further research is needed to compare and optimize different treatment strategies. Owing to the low incidence of CVST, randomized controlled clinical trials with a large sample size to explore the safety and effectiveness of MT are scarce. In addition, devices specifically designed for cerebral venous sinus and effective endovascular therapies are currently not well-established. This article summarizes different endovascular instruments and multimodal therapies for cerebral venous thrombosis. We also discuss the limitations, prospects, prognostic factors, and applications in special cases of interventional thrombectomy.

## Introduction

Cerebral venous sinus thrombosis (CVST) is a rare type of stroke, which accounts for 0.5%-1% in all stroke patients, with an estimated incidence of >10 per million per year^1^, ^2^. Common clinical symptoms include headache, seizures, altered consciousness, blurred vision, papilledema, exophthalmos, nystagmus, and focal neurological damage caused by cranial nerve palsy^3^, which are difficult to distinguish from other nervous system diseases. Common risk factors for cerebral venous sinus thrombosis include pregnancy or birth control pills, estrogen therapy, thrombophilia (especially antithrombin III deficiency, protein C and S defect, factor V Leiden or factor II thrombin prothrombin mutation), obesity, autoimmune diseases, blood hypercoagulability caused by inflammatory disease, head trauma, local infection and cancer^1^, ^4^.

**Figure 1:**
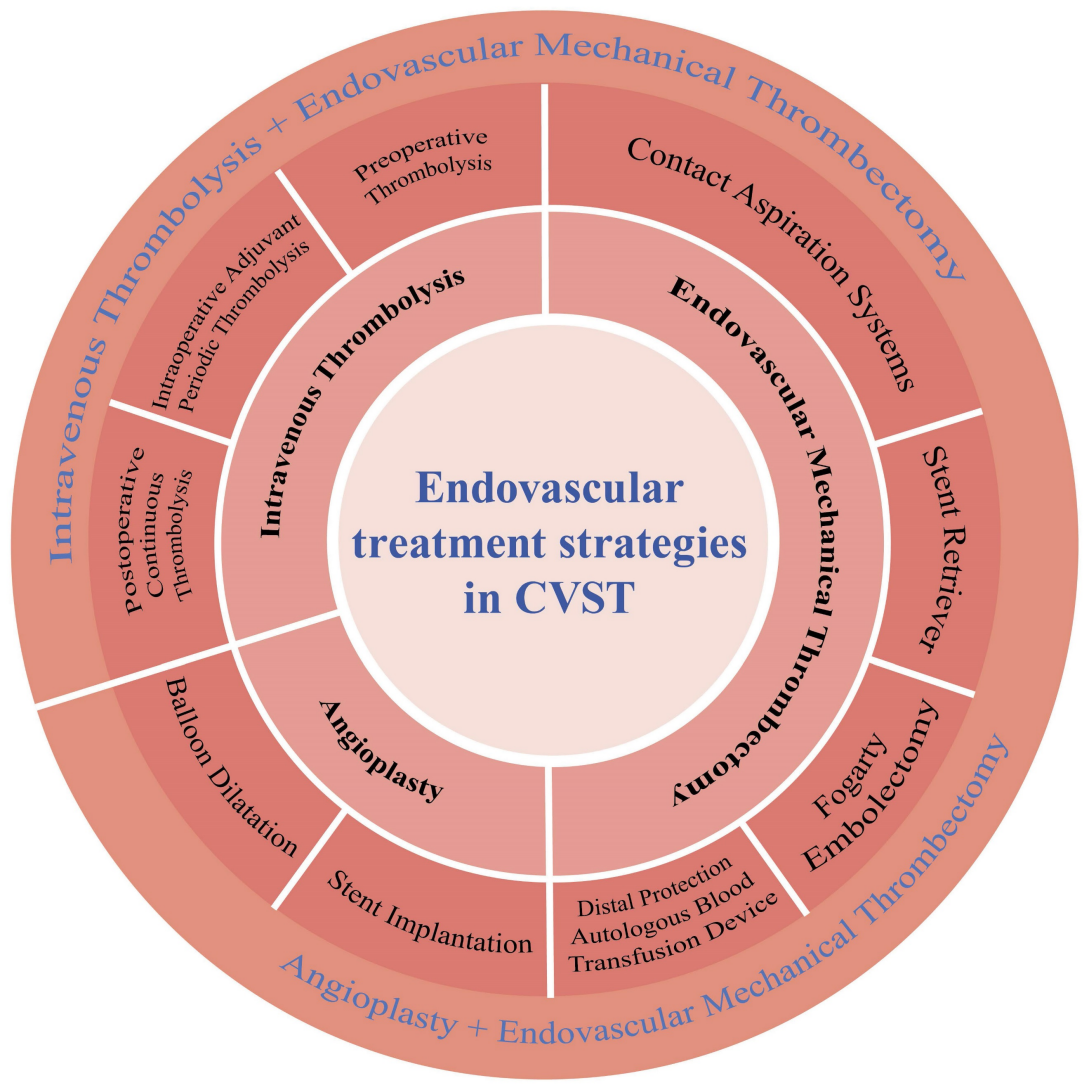
Endovascular instruments and multimodal treatment strategies for cerebral venous sinus thrombosis. CVST, cerebral venous sinus thrombosis.

Anticoagulant therapy is currently the first-line treatment for CVST. However, anticoagulants alone cannot always gain sinus recanalization. Therefore, patients who fail to respond to standard treatment or whose symptoms worsen after standard treatment often can be good candidates for endovascular therapy. Endovascular treatments mainly include local intrasinus thrombolysis (LIST), mechanical thrombectomy (MT), balloons, and stents (Fig. 1). Interventional thrombectomy is commonly performed using different types of catheters, balloons, and stents to break and aspirate the thrombus formed in the acute phase and achieve vascular recanalization. This treatment method has broad clinical applications^5^-^9^.

Since anticoagulant therapy carries the risk of expanding the bleeding range in CVST patients with intracranial hemorrhage, endovascular therapy may be potentially used as a second-line choice for those patients^10^. The American Heart Association/American Stroke Association (AHA/ASA) guidelines recommend endovascular therapy for patients whose symptoms worsen despite adequate anticoagulant therapy, or those who are at risk of intracranial hemorrhage which could lead to significant space-occupying effects^11^, ^12^. The International Study on Cerebral Vein and Dural Sinus Thrombosis (ISCVT) showed that approximately 30% of patients with one or more poor prognostic factors had poor prognosis after anticoagulation therapy^13^. Therefore, these patients may be eligible for endovascular therapy. In addition, some small preliminary studies published during the coronavirus disease 2019 (COVID-19) pandemic have shown that vaccine-induced CVST cases may be at high risk of poor prognosis and are often irresponsive to general medical management^14^, suggesting that MT may be beneficial for this patient subset^15^, ^16^. However, it is unclear whether the above predictors of poor prognosis for non-endovascular treatment also apply to MT.

A systematic review of 185 patients who underwent MT showed that the mean recanalization rate (partial or complete) was 95%^17^, suggesting that MT is safe and effective, however, the included studies were all observational and had certain bias. Recently, several studies have suggested that the curative effect of endovascular treatment is superior to systemic anticoagulation.

**Figure 2:**
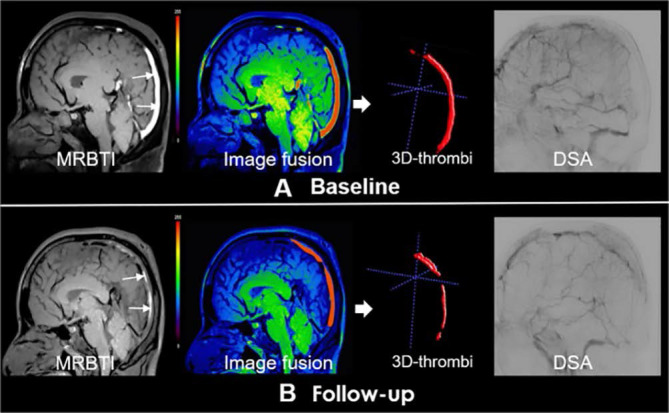
Partial recanalization of a 40-year-old male cerebral venous sinus thrombosis patient underwent endovascular treatment. Subacute clot signs in the superior sagittal sinus (SSS) and straight sinus indicated subacute thrombus (white arrows). Follow-up magnetic resonance black-blood thrombus imaging (MRBTI) image depicted partial recanalization (red arrows). Digital subtraction angiography (DSA) showed partial recanalization of SSS. Reproduced with permission from Wiley publisher (journal citation^18^). Copyright: © 2019 The Authors.

Therefore, this article summarizes the current endovascular devices and multimodal treatment methods applied to cerebral venous thrombosis and discusses the limitations, prospects, prognostic factors, and application in special cases of interventional thrombectomy. However, due to the lack of devices specifically designed for intravenous thrombectomy, some patients may not be able to achieve recanalization after thrombectomy (Fig. 2)^18^. Currently, MT is mostly used as a rescue treatment for CVST, and there is no evidence to support its use as routine treatment for CVST. Further developments in this field are required.

## Endovascular devices for CVST

### AngioJet System

In 1996, Dowd *et al.* used AngioJet catheter thrombolysis combined with venous sinus thrombolysis for the first time and achieved good clinical recovery^17^. The AngioJet mechanical thrombectomy system is a non-contact mechanical thrombectomy system, which consists of three main components: a disposable catheter, a disposable pump set, and a reusable driving unit. According to Bernoulli's principle^19^, an acute thrombus is removed by moving the microwire back and forth, injecting normal saline, and crushing and adsorbing thrombus segment by segment. The AngioJet mechanical thrombectomy system can be used alone or in combination with balloon angioplasty. A limitation of the AngioJet System is that the instrument is large and rigid, making it difficult to pass through tortuous blood vessels, especially in pediatric patients. In addition, it is unable to eliminate local lumen stenosis and dilate organic stenosis^20^, ^21^, and can easily cause perforation of the venous sinus, bleeding, and hemodilution. Studies have shown that only 55% of patients achieve complete recanalization after AngioJet device treatment, which is accompanied by a high risk of complications, thus it has been less frequently used in clinical for CVST.

### Penumbra System (Penumbra Inc, Alameda, CA, USA)

The Penumbra system is an advanced new equipment. Compared with AngioJet System, Penumbra system has better flexibility, smaller volume and superior manipulation, and it is easier to reach the distal target blood vessel through the tortuous vein, and quickly destroy the thrombus^17^. It is mainly composed of a catheter connected to a negative pressure vacuum system, which allows distal catheter insertion and continuous aspiration of thrombus fragments without causing damage to the vessel wall, and it is not necessary to remove it completely after the operation^13^. However, unlike the tough dural venous sinus, the cortical vein lacks a smooth muscle layer of the artery, thus it should be prohibited to enter the cortical vein to aspire the thrombus^22^.

As the name suggests, it was originally designed for acute ischemic stroke (AIS) to save penumbra. Similar to arterial thrombi, large-diameter distal aspiration catheters are increasingly used for direct thrombus aspiration of CVST. Choulakian and Alexander first reported four CVST patients treated with a 0.041-inch Penumbra system. All patients showed good clinical efficacy and no complications^23^. A meta-analysis involving 124 studies and 486 CVST patients receiving MT showed that the implementation of thrombo-aspiration strategies significantly increased the likelihood of partial recanalization (OR 2.79), though there is no evidence that it can significantly improve clinical outcomes or reduce the occurrence of complications compared with other devices. The results tend to be better when combined with other devices^24^. The lumen diameter of the Penumbra 5Max is larger (0.054 inch) and, if combined with other treatments, it can be compatible with other microcatheters and achieve stronger thrombus fragmentation and aspiration, which speeds up recanalization of the venous sinus, although it is more difficult to navigate through the intracranial venous sinus. The catheter can also be used for plasminogen activator infusion to local tissue, which simultaneously achieves MT and drug thrombolysis, which minimizes the frequency of catheter placement^17^. The earliest experiment on the Penumbra System combined with local infusion of urokinase in Asia introduced reperfusion catheter into the posterior part of superior sagittal sinus (SSS) without a guide catheter in the transverse sinus (TS), which reduced the risk of venous sinus injury and perforation. In addition, the large-bore catheters have good aspiration effect, therefore, some auxiliary operations such as balloon dilation can be omitted, improving the safety of the process^22^. Moreover, the new large puncture catheters (5 Max and 5 Max ACE) have a larger contact area with the thrombus and can better track, locate, and remove large thrombi^25^.

The internal diameter of cerebral venous sinus is larger than cerebral artery. As we all know, the larger diameter an aspiration catheter has, the higher aspiration efficiency it will have. Therefore, venous sinus thrombus aspiration requires aspiration catheter with larger internal diameter. In recent years, aspiration catheters with larger internal diameters, such as RED068, RED072 and SOFIA Plus, have been applied to EVT of AIS, thus their application in venous sinus need to be further studied. Among those aspiration catheters with larger internal diameter, the diameter of the SOFIA Plus catheter is 0.070 inches. Studies by Mayo Clinic and Buffalo Health Science Institute in the United States have shown that Sofia Plus can be used in AIS patients with high immediate vascular revascularization rate. Moreover, the vessel opening time can be shortened and the repeated operation can be simpler and more effective using Sofia Plus^26^. The AXS Vecta 71 and AXS Vecta74 (Stryker Neuroval, Fremont, CA, USA) with inside diameter of 0.071 inches and 0.074 inches respectively have also been approved for intracranial EVT recently^27^. Vecta 71 can be delivered through a 0.088-inch or larger guiding catheter and has shown better effectiveness than traditional suction catheters in clinical applications^28^. The Vecta 74 catheter uses the same design and materials as the Vecta 71 and must be guided by a guide catheter with 0.09 inch diameter or larger. Fabio Settecase *et al.* reported the first successful application of Vecta 74 catheter in intracranial EVT^27^.

It is well known that supportability, stability, and transmissibility are important attributes of the ideal guiding catheter in intracranial EVT^9^. However, traditional guiding catheters could not meet these three requirements at the same time. In clinical practice, the complex instrument combinations aren't safe considering patients' poor vascular conditions sometimes. The Wahoo Guide Catheter (Q 'Apel Medical, Fremont, CA) was approved by the Federal Drug Administration (FDA) in 2018. It uses "SelectFlex technology" at the distal end so that it can alternate between "tracking" and "support" modes as needed during operation. In other words, it can be "soft" or "hard", thus reducing the need to use multiple catheters in complex surgeries, adapting to complex and changeable vascular conditions, and building stable and reliable access. The study by Waters MJ *et al.* showed that the Wahoo catheter system is feasible in a variety of intracranial EVT surgeries, and can be flexibly used in tortuous vessels^29^. Moreover, Armadillo is also a new guide catheter similar to the Wahoo Guide Catheter, using the same "SelectFlex technology", it has the same advantages, and specialize in entering the neurovascular system through the radial artery. However, there is a lack of research on its application in intracranial EVT, so its safety and effectiveness need to be further verified.

### Stentrievers for MT

The Solitaire device is a laser-engraving, self-expanding and completely retrievable stent approved by the FDA for ischemic stroke caused by intracranial large vessel obstruction^30^. The SWIFT trial (SOLITAIRE FR With the Intention for Thrombectomy) proved its effectiveness in treating ischemic stroke^31^. The Solitaire stent can be retrieved (up to three times), with good plasticity and controllability. The device embeds the thrombus in the stent and removes it. This process not only dislodges the thrombus attached to the blood vessel wall, but also fragments the thrombus, thereby facilitating contact between the thrombus and thrombolytic drugs^32^, ^33^ Moreover, endovascular chemical thrombolysis can be optional in some cases because of the large vascular channels created by the Solitaire device^31^.

A study using Penumbra thrombus aspiration system combined with Solitaire FR device for the treatment of CVST showed good clinical recovery^34^, ^35^. The Solitaire stent can be used as an anchor when the aspiration catheter aspirates the thrombus at distal access. Furthermore, the Solitaire stent and thrombus can enter the large proximal guide sheath as a whole under continuous aspiration, to achieve thrombus aspiration and stent withdrawal at the same time^36^. In addition, it is difficult for Solitaire stent thrombectomy alone to achieve recanalization in the treatment of CVST, therefore, contact thrombolytic therapy must be combined, particularly for CVST with a heavy load. This program significantly shortens the duration of thrombolytic therapy, reduces patients pains, and reduces the incidence of complications. However, severe patients, especially those at risk of cerebral hernia, still have a poor prognosis after thrombectomy. Compared with the AngioJet and Penumbra thrombus aspiration systems, Solitaire is more useful in the sigmoid and transverse sinuses. The advantages of Solitaire are that it can be easily manipulated in the venous sinus, firmly attached to the thrombus, and can be used repeatedly to quickly recanalize the venous sinus. In addition, a special Solitaire device needs to be applied at the sinus confluence with a large angle to reduce the risk of accidental detachment^31^. A recent meta-analysis showed that the implementation of stent retriever significantly increased the likelihood of partial recanalization (OR 1.76), significantly reduced the risk of death (OR 0.371). and significantly improved clinical prognosis at last follow-up compared to other thrombectomy devices (OR 2.588)^24^. However, owing to the limited sample size of the study, it was difficult to draw a definite conclusion regarding the efficacy of the Solitaire stent.

Because the Solitaire stent is designed for arterial thrombectomy, there are currently no specifications suitable for the cerebral venous sinus. This is mainly applicable to a small amount of fresh thrombus. However, the grasping and clearance abilities of this system are not ideal for subacute chronic thrombus or large thrombus, and it requires manual aspiration or combination with the Penumbra System. Moreover, the operation is complex and may cause breakage and damage to expensive instruments. In addition, the thrombus in straight sinus (SS) cannot be cleared and there is a risk of pulmonary embolism after thrombus detachment^36^.

The Trevo device has a similar structure to the Solitaire. Both are laser-engraved self-expanding stents, and the difference lies in clot integration techniques^37^. The Trevo stent has vertical radial struts that allow effective thrombus integration. Due to its flexible design, studies have shown that Trevo has smoother vascular navigation and can navigate to more distal vessels, and it has been approved by the FDA for early reperfusion therapy in patients with AIS^38^. TREVO 2 trial (Thrombectomy Revascularization of Large Vessel Occlusions in Acute Ischemic Stroke) and TRACK real-world study (The TREVO Stent-Retriever Acute Stroke Registry) have confirmed efficacy and safety in the treatment of AIS^39^, ^40^. Justin R Mascitelli *et al.* first reported thrombectomy for cerebral venous sinus pyogenic thrombi using a novel combination of Trevo stent retriever and penumbra ACE aspiration catheter^41^. Due to large and long-segment thrombus in the SSS, SS, and bilateral TS, in specific procedure, with the Trevo as an anchor in the superior sagittal sinus, the ACE catheter was aspirated back and forth between the superior sagittal sinus and the left transverse sinus. The venous drainage was improved by stent anchor with mobile aspiration technique, which is especially suitable for the removal of a large amount of long-segment thrombus. However, more clinical studies are needed to confirm the effectiveness of Trevo in CVST thrombectomy.

In recent years, new thrombectomy devices with larger diameter and length may bring more options for the treatment of CVST. The CatchView (CV) thrombectomy device is a novel self-expanding, resheathable, closed-cell design, nickel-titanium cutting stent^42^. The CVMaxi 6mm in diameter with an optional 50mm length is not available in many other stent retriever devices and may be able to show advantages in CVST thrombectomy. However, although current clinical studies suggested that CV achieved higher reperfusion rate and good prognosis rate compared with standard stent retriever in acute arterial ischemic stroke^43^, there is still a lack of studies to show its safety and effectiveness in CVST. The Tigertriever XL device is a novel, fully visualized, operator-adjustable stent retriever with maximum length of 53mm that can be extended to a diameter range from 1.5 up to 9mm^44^, which corresponds to the sinus diameters of the cerebral venous system, 9mm for the internal jugular vein, 6.4 to 6.5mm for the transversal-sigmoid sinus, and 3.5 to 5.7mm for the superior sagittal sinus^45^, ^46^. The TIGER (Treatment with Intent to Generate Endovascular Reperfusion) study showed that the Tigertriever device was highly effective and safe in patients with large vessel occlusive stroke compared with the Trevo and Solitaire devices^47^, but studies are lacking in CVST.

### Fogarty embolectomy catheters

Fogarty embolectomy catheters are suitable for removing fresh soft artery thrombus^5^, and also for removing fresh cerebral venous sinus thrombus. The advantages of the Fogarty embolectomy catheters include the following: it can remove large fragments of thrombus and form channels within the thrombi. Balloon dilation can crush a thrombus and improve lumen stenosis via balloon angioplasty. Fogarty embolectomy catheters are mostly used to assist other devices in thrombus removal. The disadvantage of Fogarty embolectomy catheters is that they cannot effectively remove non-acute or large thrombus, and needs to be used with other devices. Moreover, the operation is complex, and the catheters block blood flow and interfere with the venous sinus wall and sinus pressure. Fogarty embolectomy catheters can be combined with Penumbra system and local drug thrombolysis^36^. In 2000, Novak *et al.* first used Fogarty embolectomy catheters to remove thrombus in both the transverse and the sagittal sinuses, which achieved complete symptomatic relief. However, some studies have achieved thrombus removal using Fogarty balloon catheter after continuous infusion of urokinase, but can only partially clear the thrombus, accompanied by the risk of pulmonary embolism and subdural hematoma^5^. In general, the safety and effectiveness of Fogarty catheter in CVST need to be confirmed, and clinicians should be cautious when choosing this device for CVST.

### Distal protection device (Angioguard, Cordis, Inc) and autologous blood transfusion device (Autolog, Medtronic, Inc)

The principle of a distal protection device and an autologous blood transfusion device is mechanical thrombus breakage combined with negative pressure suction, which causes little damage to the vessel wall. Autologous blood transfusion can be achieved by thrombus suction of the autologous blood transfusion device using negative pressure suction to avoid excessive blood loss. However, the operation is long-lasting and complex, and cannot completely remove subacute, chronic, or large thrombi. The quality of recanalization is poor, and umbrella damage and fractures may occur during surgery^48^.

The aforementioned interventional thrombectomy devices differ in principle, and each has its advantages and disadvantages. Table 1 presents a comparison of the different device.

## Multimodal Therapy for CVST

At present, most devices used in CVST are designed for arterial stroke, and the aim of cerebral venous sinus thrombectomy cannot be achieved using single device. Currently, multi-modal treatment strategies are widely adopted in clinical practice.

### EMT combined with LIST

Intrasinus thrombolysis includes pre-thrombolysis, intraoperative adjuvant periodic thrombolysis (IOT), and postoperative continuous thrombolysis (CTI), and is especially suitable for short-term surgery and dissolution of thrombosis in the cortical, bridging, and other small veins. Current international guidelines recommend that all rt-PA available patients with emergent arterial large-vessel occlusion undergo intravenous thrombolysis (IVT) before accepting thrombectomy^49^, ^50^, however, IVT cannot delay the implementation of the MT. MT is also suitable for patients ineligible for IVT, so IVT and EMT MT play complementary roles in the treatment of patients with acute stroke^51^, ^52^.

Postoperative continuous thrombolysis is also very important to remove residual thrombus and prevent postoperative re-occlusion. One study showed that although the application of the second-generation stents can achieve complete recanalization of venous sinus in angiography, bridging cortical vein thrombosis and sinus thrombosis can be detected on optical coherence tomography (OCT), which explains the success of MT, but with poor clinical outcomes. Therefore, chemical thrombolysis should be performed after sinus surgery to dissolve the remaining blood clot^53^. MT combined with local drug thrombolytic therapy can be used as a rescue treatment strategy when acute venous hypertension needs to be urgently relieved in patients with worsening condition after drug anticoagulant therapy^54^, For severe patients with lesions involving multiple venous sinuses, computed tomography (CT) and magnetic resonance imaging (MRI) should be combined to analyze the obstructed sinuses before thrombolysis can be performed^1^, ^55^. Recanalization during thrombectomy is unnecessary subsequent heparinization and local continuous thrombolysis can gradually recanalize the venous sinus^56^. Penumbra thrombosis suction system can not only reconstruct venous blood flow but also create a channel for subsequent venous thrombolysis^22^.

Some studies have used various devices, such as Fogarty catheter, balloon angioplasty, or vascular ejection system, to destroy or remove thrombus, and then local thrombolytic infusion was used to treat CVST patients. These studies demonstrated good efficacy^17^, ^57^, ^58^.

In a retrospective study, recanalization rates, prognosis, surgery-related complications, sequelae, and postoperative bleeding rates were compared among MT plus IOT, MT plus CTI, and MT plus IOT plus CTI for CVST. The results showed that local thrombolysis combined with EMT was relatively safe, and there were no significant differences in efficacy and safety between the three schemes. The recanalization rate and postoperative bleeding rate in 82 patients were 82% and 4%, respectively^59^. However, further randomized controlled studies are required to determine the optimal treatment scheme. In addition, a report on both AngioJet catheter thrombolysis and transarterial thrombolysis provides a new option for patients with extensive dural sinus or cerebral venous thrombosis and progressive neurological deterioration^60^.

In a retrospective study involving 185 cases, 7% of patients receiving MT alone and 11% of patients receiving MT combined with LIST at the same time experienced postoperative bleeding, but the difference was not statistically significant^17^. In 2019, Lewis *et al.* conducted a systematic review and meta-analysis that included 116 patients undergoing intraarterial/intrasinus chemical thrombolytics combined with mechanical thrombolysis, the complete recanalization rate was 75%, and the postprocedural hemorrhagic rate was 17%^61^. A study comparing MT combined with LIST and LIST alone did not show a significant difference in efficacy^58^. However, this was a non-randomized retrospective study with a large bias. Future research needs to compare the risk of bleeding complications between CVST treated with MT alone and MT combined with LIST, especially in cases of bleeding before treatment.

### Multiple mechanical thrombectomies combined with catheter aspiration

There is insufficient evidence to determine which MT regimen provides the best outcome. In many cases, a combination of multiple therapies is required to reduce the amount of thrombolytic drugs required and the risk of bleeding. Currently, many thrombectomy methods are used in combination with large-lumen suction catheters. Radoslav Raychev e*t al.* first performed aspirational thrombectomy in patients with malignant cerebral edema using a maxPenumbra thrombectomy catheter combined with Solitaire FR thrombectomy device and achieved complete recalculation of occluded sinus. This combined procedure has also been successfully used for cerebral venous sinus thrombosis in children. The 5Max catheter can be positioned at the thrombus, thus enabling continuous catheter aspiration during Solitaire FR thrombectomy^62^. It also has the advantages of thrombus aspiration and removal of large thrombus using stents, thereby maximizing the recirculation rate and reducing the risk of pulmonary embolism. Compared with all previously reported intravascular therapies, this approach prevented the development of malignant edema and avoided invasive surgery, and was faster, safer, and more effective. However, this combined regimen could not replace systemic anticoagulation, and only served as a supplement^62^. In addition, Fogarty percutaneous balloon catheter thrombectomy can be combined with Penumbra Aspiration system. The balloon is placed in the affected sinus or the distal end of the thrombus in the sinus for anchoring, and then inflated and inhaled into the thrombectomy system or guided by the retraction of the sheath to complete thrombectomy^36^. However, whether these two regimens should be further combined with thrombolytic therapy remains controversial. In addition, stent thrombectomy and AngioJet devices combined with the Penumbra thrombosis system have been reported^62^-^66^. A retrospective study analyzed 14 refractory severe intracranial hemorrhage complicated with venous sinus thrombosis (SH-CVST) patients treated with tandem double stent retriever combined with intermediate catheter aspiration. Double stent retrievers were placed in tandem, with the second stent retriever placed caudal to the first stent retriever. Both stent retriever devices combined with intermediate catheter aspiration were pulled back. Among 14 patients, 10 patients were successfully recanalized and 11 patients had modified Rankin scale (mRS) scores of 0-2 at 12 months postoperatively, which suggests that mechanical thrombectomy combined with aspiration is a potentially effective treatment strategy for refractory SH-CVST^67^. A recent comprehensive meta-analysis, encompassing 55 patients sourced from four retrospective studies, has meticulously evaluated the safety and efficacy of utilizing stent-retrievers in conjunction with catheter aspiration in the refractory CVST. The findings revealed that 36% of patients achieved complete recanalization, while a good clinical outcome was observed in 72% of patients. Notably, the incidence of adverse events, including hemorrhagic, ischemia, and neurological complications, ranged modestly from 0 to 7%^68^. In general, the combination of stent retrievers and catheter aspiration emerges as a pivotal therapeutic approach for rescuing patients afflicted with refractory CVST. Nevertheless, the limited number of studies incorporated into this analysis introduced significant heterogeneity in the results, underscoring the pressing need for further deliberation through standardized, randomized controlled trials. To optimize clinical outcomes, it is imperative to devise individualized, combined treatment strategies tailored to the unique characteristics of each CVST patient.

### MT combined with stenting

Currently, using MT devices designed for arterial stroke are unable to effectively remove a large amount of clot from the great venous sinus and achieve recanalization in all patients. In addition, although the occluded venous sinus was recanalized after MT in some patients, the stenosis was not completely relieved, or the external pressure of the local venous sinus was not significantly reduced in a short time owing to local brain edema and intracranial pressure elevation, resulting in the formation of thrombosis in a short time after the recanalization of venous sinus. Therefore, after MT for local thrombosis, stent implantation can effectively achieve venous sinus recanalization and avoid venous sinus re-occlusion in cases of contrast agent retention. Current studies have confirmed that stenting (combined with balloon angioplasty) can be used as a rescue strategy when traditional thrombectomy methods fail^69^. However, there are still limited number of studies on stent implantation or angioplasty as the first-line intravascular treatment for CVST.

## Application of interventional thrombectomy in special populations

### CVST for children

Although the incidence of CVST in children is low, it has recently increased^70^. Owing to the incomplete development of collateral circulation in the cerebral venous system and lack of compensation in pediatric patients, anticoagulant therapy is not suitable. In addition, 17 observational studies including 1200 pediatric patients showed that thrombolytic therapy has some positive effects, but the level of evidence is very low. As patients receiving thrombolytic therapy may suffer from disease deterioration, experts suggest that thrombolytic therapy is not recommended for pediatric patients without cerebral ischemia^71^. Recent studies have shown that MT is suitable for children with CVST who do not benefit from conventional treatment. This retrospective study analyzed the clinical and radiographic data of seven pediatric patients with CVST at five centers treated with MT over the past 10 years. The mRS scores of six patients 90 days after surgery were 0 or 1, indicating good efficacy, and only one patient had neurological impairment, suggesting that timely MT may be safe and effective in severe pediatric patients with poor imaging or clinical recovery after anticoagulation therapy^72^. Another study performed emergency repeated balloon angioplasty combined with catheter aspiration in three comatose pediatric patients and found that the SSS was completely recanalized, and there was no neurological impairment at discharge, suggesting that MT should be performed as soon as possible for comatose pediatric CVST patients^73^. In addition, thrombosis in the neonatal period accounts for a large proportion of cerebral venous sinus thrombosis in children, whereas the incidence of neurological dysfunction is high in neonates treated with low molecular weight heparin (especially in cases with multiple sinus thrombosis)^74^. A case report on MT in the treatment of cerebral venous multi-sinus thrombosis in neonates suggested that MT is also suitable for the treatment of neonatal CVST, and it was reported that neurological prognosis improved after MT^75^.

### Patients with intracranial hemorrhage or high risk of intracranial hemorrhage

Although anticoagulation is the first-line treatment for deep CVST, intracranial hemorrhage caused by anticoagulation and subsequent permanent neurological dysfunction are common. A meta-analysis of 120 patients from 69 studies has led to similar conclusions. Therefore, early MT may be suitable for patients at high risk of intracranial hemorrhage^10^.

**Figure 3:**
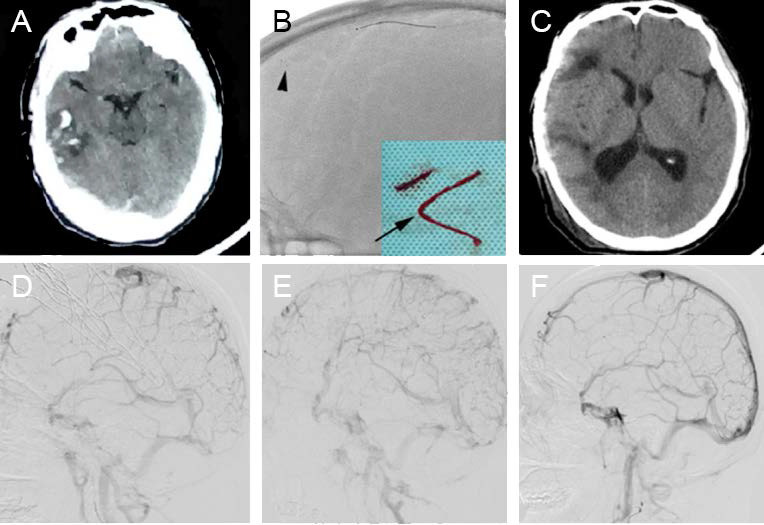
(A) A 47-year-old male patient presented with a headache and worsened in the next day. The computed tomography (CT) scan revealed right temporal lobe hemorrhage. (B) Stent retriever thrombectomy was performed. The arrow indicates the Solitaire FR stent deployed in the occluded superior sagittal sinus (SSS). The arrowhead indicates the clots that were removed. (C) The 4-month follow-up CT scan was normal, apart from the cranioplasty. (D) The lateral digital subtraction angiography (DSA) sinus phase image of the right side demonstrated extensive severe cerebral venous sinus thrombosis. (E) DSA revealed partial recanalization of the occluded sinus. (F) DSA revealed complete recanalization of the SSS with normal arteriovenous blood flow in the left transverse sinus at the 4-month follow-up. Reproduced with permission from Spandidos publisher (journal citation^76^). Copyright: © Wang et al.

SH-CVST can cause massive hematoma, edema, and cerebral hernia, which can be fatal. Studies have shown that intracranial hemorrhage before treatment can lead to poor functional prognosis, higher incidence of permanent injury, increased risk of intracranial hemorrhage and higher mortality^10^. Under the circumstances, anticoagulation or thrombolytic therapy alone may further aggravate the hematoma. A retrospective study including eight patients who underwent stent retriever thrombectomy combined with long-term local thrombolysis (SRT-LLT) after unsuccessful intravenous anticoagulation between 2013 and 2018 achieved angiography-confirmed successful recanalization in all patients, with no treatment-related complications or deaths. Figure 3 shows an image of a patient who achieved hematoma absorption and edema dissipation after venous sinus thrombectomy (Fig. 3) 76. This study showed that SRT-LLT is a feasible, safe and effective treatment regimen for SH-CVST, and can be used as a rescue treatment for specific SH-CVST patients^76^. A retrospective study was conducted to analyze the clinical data of 56 patients with SH-CVST admitted for 9 years, and all patients received LIST with or without MT. The complete recanalization rate was 67.8% and the good prognosis rate at discharge was 87.5%. The mRS score was 0-2 in 49 of the 51 patients who were followed up at 6 months after surgery. These results suggest that endovascular treatment may improve the clinical outcomes of most patients with SH-CVST, however, this needs to be confirmed in prospective controlled studies^77^.

### CVST secondary to trauma

Acute cerebral venous sinus thrombosis caused by trauma can lead to malignant cranial hypertension, which does not respond to drug therapy or surgical decompression, leading to serious consequences. However, there is no consensus on the treatment strategies for those cases. A patient with post-traumatic cerebral venous thrombosis suffered increased intracranial pressure after hemicranial decompression, and then underwent dural sinus thrombectomy and stent implantation, resulting in immediate relief of intracranial hypertension and a good prognosis^78^. This case suggests that MT combined with stenting is effective in recanalizing and reducing intracranial pressure in CVST secondary to trauma.

### Deep cerebral venous system thrombosis

Previous studies have shown that MT can lead to a smaller deep cerebral vein or straight sinus perforation^58^. However, the development of new surgical instruments has largely eliminated these limitations. MT for deep cerebral venous system thrombosis has rarely been reported, and the reported cases are usually successful. In addition, endovascular therapy is often used as salvage therapy in patients with poor prognosis after medical treatment, thus the results are often biased in the direction of poor outcomes.

## Prognostic factors

Salottolo *et al.* suggested that hormone-related etiology, migraine, and brain edema are indicators of good prognosis for CVST endovascular treatment^79^. In a prospective study conducted by Li *et al.*, magnetic resonance black blood thrombus imaging (MRBTI) was performed on patients before endovascular treatment, and the thrombus signal-to-noise ratio, thrombus formation time and baseline thrombus volume were compared between complete and partial recanalization groups. This study showed that acute thrombotic signs on MRBTI were associated with complete recanalization. MRBTI is a reliable method for determining thrombus composition and can be used to select the most suitable patients for endovascular therapy^18^. In the future, advanced minimally invasive imaging and diagnostic techniques will be required to accurately measure the thrombosis time of CVST.

## Limitations

### Unclear criteria for prognostic evaluation, efficacy, target patient cohorts, and time-to-treatment

There is no consensus on how to define a successful implementation of MT and no uniform clinical outcome indicator. In contrast to AIS, partial recanalization may improve symptoms because the residual thrombus can be dissolved by autologous fibrinolysis after MT^80^. Therefore, the extent of recanalization did not affect clinical outcomes. In addition, CVST patients are younger, and the prognostic indicators for AIS, such as a mRS score, may not be suitable for CVST. Due to the variety of clinical images in patients with CVST, the criteria for MT assessment by imaging have not yet been established.

Most studies did not analyze different patient groups or classify patients according to the location of occlusion^81^, therefore, patients suitable for MT could not be screened. It is speculated that sometimes no obvious effect was achieved in MT possibly because the patients did not meet the selection criteria.

Regarding the timing of MT therapy, in a recent study, Mohammaddian *et al.* defined ineffective anticoagulation as worsening of symptoms (in patients with more than one venous sinus) after at least 4 days of adequate anticoagulation with heparin^82^. There is no clear definition of ineffective anticoagulant therapy, the duration of anticoagulant therapy is unknown until it fails and endovascular therapy is performed. Studies have shown that over time, the resistance of thrombus to fibrinolytic drugs increases, and the chances of successful MT may decrease (particularly with adjunctive local intravenous thrombolysis)^83^. In contrast to AIS, the period during which a thrombus exists before diagnosis is uncertain, renderings the timing of MT treatment even more difficult to determine.

### Lack of selection criteria for therapy options

In a small retrospective series study, Soleu *et al.* found MT to be superior to LIST with a lower risk of bleeding, but some studies advocate the use of LIST in milder conditions^58^. One study has shown that due to the tiny size of deep brain veins and the high risk of vascular perforation, pharmacological thrombolysis should be preferred over MT^25^. However, MT is only used as a rescue therapy in rare cases. In addition, different MT strategies have been used in different centers, and there is no uniform treatment or combined regimen. Current systematic reviews indicate that no study has shown the superiority of one particular EVT technique over another^59^, ^61^, ^84^.

### Safety and effectiveness remain to be proven

Available data in CVST patients do not support MT as a routine treatment. European guidelines do not recommend endovascular therapy in patients with acute cerebral venous thrombosis at a low risk of poor prognosis^85^. In addition, no studies have shown a high recanalization rate for MT, and some studies do not support its safety and efficacy^86^, ^87^. Currently, the only randomized controlled trial of CVST treatment (Thrombolysis or Anticoagulation for Cerebral Venous Thrombosis Study, TO-ACT) suggested that endovascular treatment may not improve the functional prognosis of CVST patients^86^. However, there are some limitations to this study, such as small sample size, lack of uniform time window from the onset of CVST to treatment, different inclusion criteria, and treatment methods in different clinical centers. In addition, the study had limitations in evaluating veins with the method of assessing arteries, which biased the results. Other studies did not support the use of MT or thrombolytic therapy in patients with acute cerebral venous sinus thrombosis^17^. In an analysis of a national inpatient sample from 2004 to 2014, mortality was higher in patients who received endovascular therapy^88^. However, MT is often used after anticoagulation failure, and intravascular devices are only indicated for thrombosis in larger vessels (SSS, TS, sigmoid colon, and SS) with a high risk of poor prognosis even if other current state-of-the-art treatments are used, and that existing venous MT has not been optimized and is used only as salvage therapy in rare cases, so these studies that do not support the safety and effectiveness of MT in CVST patients may be biased^89^.

Randomized clinical trials comparing the efficacy of intracerebral venous sinus thrombolysis or MT with standard anticoagulant therapy are lacking. Due to the limited number of patients treated with each endovascular treatment technique, it is difficult to make a comprehensive comparison between the different techniques.

### Post-operative complications

Tedious and lengthy MT procedures lead to a higher incidence of complications in complex cases. Although some small non-randomized studies and case reports have shown recanalization rates of 70-90% with endovascular therapy, the incidence of concomitant intracranial hemorrhage is approximately 10%, and fragile cortical veins cannot be recanalized because of the risk of vascular perforation. The venous sinuses and bridging veins at the junction site are also more vulnerable. The most serious complication resulting from vascular perforation is new intracerebral hemorrhage, the incidence of which is higher than that of anticoagulant therapy^17^, ^84^. In a comparative study between MT and LIST, treatment-related complications occurred more frequently in the MT group^58^. For example, AngioJet devices can lead to fluid overload. Catheter damage to the vascular endothelium increases the risk of iatrogenic thrombosis, and thrombotic debris during MT can lead to pulmonary embolism^13^. Compared with other endovascular therapies, MT is also associated with surgical site complications, including hematoma, soft tissue infection, iatrogenic arteriovenous fistula formation, peripheral nerve injury, and the risk of perioperative anesthesia^5^.

### Techniques and equipment remain to be improved

Devices currently used for endovascular treatment of CVST are designed for AIS, however, the mechanism and composition of arterial and venous thrombosis are different. Arterial thrombosis is caused by the ulceration of atherosclerotic plaque, platelet activation, and a hypercoagulable state. It is primarily composed of platelets and small amounts of fibrin. Venous thrombosis is mainly caused by blood stasis and a hypercoagulable state, therefore, the thrombosis shows only slight adhesion to the vessel wall and breaks off easily. It is mainly composed of fibrin and red blood cells. In addition, compared with cerebral arteries, the walls of the cerebral veins lack a smooth muscle layer and have no valves. Because the reflux of cerebral venous blood converges mainly into the dural venous sinus, the diameter of the venous sinus cavity is large and the thrombus burden is high, so the specifications of existing devices are generally not applicable to the cerebral venous sinus^90^. A preclinical animal model using a second-generation stent retriever showed that the arterial thrombectomy device is not suitable for cerebral venous sinus thrombosis.

AIS stent retrievers and aspiration catheters are usually smaller in diameter than the average diameter of the SSS in the occipital region (approximately 10 mm) and cannot completely remove the thrombus^91^. The Fogarty catheter system is sufficiently large; however, its stiffness does not apply to smaller sinuses or more distal vessels. In addition, venous sinuses connecting cortical veins vary in size, and venous sinuses at the crossing site may be damaged by current stent retrievers^54^. Therefore, further development of endovascular devices specifically designed for the cerebral venous sinuses is required to achieve better therapeutic outcomes.

## Conclusions

For patients with severe CVST who are refractory to anticoagulant therapy, have massive thrombus burden, involve the SSS, and are in the acute phase, interventional thrombectomy is a rescue treatment strategy. Numerous clinical trials and case studies have shown promising application prospects. This article reviews the indications, prognosis, and existing problems and compares many different endovascular treatment strategies and multimodal treatment options. Currently, stent retriever thrombectomy combined with intermediate catheter aspiration is widely used in clinical practice, and studies have suggested a favorable recanalization effect. For patients with venous sinus thrombosis and localized sinus stenosis, mechanical thrombectomy combined with stent implantation is an effective treatment. The appropriate combined treatment strategy should be selected according to the individual situation of the patient, including the location and burden of thrombi. However, the devices currently used for interventional thrombectomy in CVST are designed for AIS^54^, and may lead to sinus rupture and other catheter-related complications. Further development and testing of a new generation of devices specifically designed for venous thromboembolism therapy are urgently needed in the future. Further clinical trials are required to better assess the duration of adjuvant anticoagulant therapy, and the selection of endovascular treatment options and follow-up procedures should be improved. Studies of the pathomechanisms of cerebral venous thrombosis will also contribute to the development of optimal endovascular guidelines. In addition, it should be noted that different endovascular treatment strategies are applicable to different CVST patients and clinical conditions, and specific prognostic and curative effect indicators should be established to perform MT for CVST patients, and then patients should be grouped to select the most appropriate treatment strategy.

Since severe CVST is uncommon, in the future, it is necessary to prospectively collect case information through international collaboration using public data to provide further evidence to study the safety and efficacy, surgical methods, and treatment options of CVST and to develop interventional devices specifically designed for the cerebral venous sinus.

## Figures and Tables

**Table 1:** Comparison of major thrombectomy devices for intravascular treatment of cerebral venous sinus thrombosis

Devices	Mechanism	Advantages	Disadvantages	Possible Complications
**AngioJet** (MEDRAD, Inc, Warrendale, PA, USA)	A rheolytic thrombectomy system which sprays normal saline, breaks and adsorbs the thrombus segment by segment	Effective for acute thrombus	The instruments are heavy and rigid, making it difficult to get through the tortuous vessels Low penetration rate (55%) Local lumen stenosis could not be eliminated	Cause damage to blood vessels Too much fluid injection can dilute the blood
**Penumbra thromboaspiration system** (Penumbra,Inc, Alameda, CA, USA)	It's a new device that sucks out blood clots	It is relatively compliant and easy to reach the distal end through tortuous vessels. It's easy for large lumen 5MAX, 5MAX ACE to aspirate thrombus	Non-acute thrombus or thrombus load cannot be effectively removed; Recanalization failure Heavy load thrombus obstructs the catheter, which needs to be removed and repeatedly rinsed, and repeatedly inserted, which is complicated	
**Stent retriever** Solitaire FR (Covidien, Irvine, CA, USA)	New thrombectomy device, the stent can be recovered after thrombectomy	Repeatable application (up to 3 times), using stent to capture thrombus; Combined with Penumbra can improve clearance	The device is designed for arterial thrombectomy and there is not suitable size for cerebral venous sinus; the grasping and removing ability is poor for subacute chronic thrombus or large thrombus; Requires manual suction or Penumbra combination with complex operation and high cost; Can't clear straight sinus thrombosis	Risk of pulmonary embolism The instrument was broken during operation
**Fogarty Embolectomy**	Balloon catheter is suitable for crushing and removing acute fresh thrombus. The thrombus was crushed by balloon expansion; Angioplasty to improve lumen stenosis	Assist with other devices to clear the clot	Non-acute thrombus or large thrombus cannot be effectively broken and cleared Need to cooperate with other devices to remove thrombus, the operation is complicated Block the blood flow and interfere greatly with the wall of venous sinus and the pressure in the sinus	Risk of pulmonary embolism and subdural hematoma
**Remote Protection Device (Angioguard, Cordis Inc)** **Autologous Blood Transfusion Device (Autolog, Medtronic, Inc)**	Mechanical thrombus crushing combined with negative pressure suction	Damage of vascular wall is small, combined with autologous blood transfusion and negative pressure suction device for thrombus extraction; autologous blood transfusion can avoid excessive blood loss	Long operating time Complex operation Subacute chronic thrombus or large thrombus cannot be completely removed, and the recalculation effect is poor	The protection umbrella can be damaged and broken

## Abbreviations

AHA/ASA: American Heart Association/American Stroke Association
AIS: acute ischemic stroke
COVID-19: coronavirus disease 2019
CT: computed tomography
CTI: continuous thrombolysis
CV: CatchView
CVST: cerebral venous sinus thrombosis
EVT: endovascular treatment
FDA: Federal Drug Administration
IOT: intraoperative adjuvant periodic thrombolysis
ISCVT: International Study on Cerebral Vein and Dural Sinus Thrombosis
IVT: intravascular thrombolysis
LIST: local intrasinus thrombolysis
MRBTI: magnetic resonance black blood thrombus imaging
MRI: magnetic resonance imaging
mRS: modified Rankin scale
MT: mechanical thrombectomy
OCT: optical coherence tomography
SH-CVST: severe intracranial hemorrhage complicated with venous sinus thrombosis
SRT-LLT: stent retriever thrombectomy combined with long-term local thrombolysis
SS: straight sinus
SSS: superior sagittal sinus
SWIFT: SOLITAIRE FR With the Intention for Thrombectomy
TIGER: Treatment with Intent to Generate Endovascular Reperfusion
TO-ACT: Thrombolysis or Anticoagulation for Cerebral Venous Thrombosis Study
TRACK: The TREVO Stent-Retriever Acute Stroke Registry
TREVO 2: Thrombectomy Revascularization of Large Vessel Occlusions in Acute Ischemic Stroke
TS: transverse sinus
